# Eat, sleep, play: health behaviors and their association with psychological health among cancer survivors in a nationally representative sample

**DOI:** 10.1186/s12885-022-09718-7

**Published:** 2022-06-13

**Authors:** Trevin E. Glasgow, Kandace P. McGuire, Bernard F. Fuemmeler

**Affiliations:** 1 Department of Health Behavior and Policy, Virginia Commonwealth University, 830 E. Main St, Richmond, VA 23219 USA; 2 Department of Surgery, Virginia Commonwealth University, 907 Floyd Ave, Richmond, VA 23284 USA

**Keywords:** health behavior, sleep hygiene, well-being, attitude to health, psychological health, cancer survivors

## Abstract

**Background:**

Cancer survivors are able to live much longer today than in the past due to advances in treatment. The promotion of health behaviors is important to address among cancer survivors. Not only can health behaviors reduce the risk of comorbidities, but they may also be key to improving psychological health among cancer survivors, such as psychological distress, perceptions of one’s general health, and distress of cancer mortality (i.e., cancer fatalism). Our overall goal is to 1) determine which individual health behaviors (e.g., fruit consumption, physical activity, and sleep) are associated with psychological health among cancer survivors and 2) determine if the number of health behaviors engaged in is associated with better psychological health among cancer survivors.

**Methods:**

Using data from the HINTS 5 Cycle III survey (*N =* 856 cancer survivors), we compared whether participants who met guidelines for seven health behaviors (e.g., fruit and vegetable consumption, moderate physical activity, sleep duration) had improved psychological health. Multiple ordinal regression models controlling for sociodemographic variables were used to determine if meeting more recommendations was related to psychological health and then specifically which behaviors were of greatest importance to psychological health.

**Results:**

Meeting guidelines for sleep quality and moderate physical activity was associated with higher general health and meeting guidelines for vegetable intake and better sleep quality was associated with less psychological distress. Although the associations were not significant, cancer survivors who met more of the health behavior guidelines reported higher general health, lower psychological distress, and less distress about what could be done to lower one’s chances to get cancer.

**Conclusions:**

Meeting the guidelines for individual and multiple health behaviors is associated with favorable psychological health among cancer survivors. The findings can contribute to the development of health behavior interventions that focus on multiple health behaviors to improve psychological health and quality of life among cancer survivors.

## Background

In 2019, the number of cancer survivors was estimated to be 16.9 million, with the number expected to grow past 22 million by 2030 [1]. As the United States lifespan continues to rise, so does the risk of cancer development, particularly among the older adult population [2]. Despite increasing incidence rates in cancer over the past 20 years, survival rates have improved due to improvements in medical treatment [1, 3]. With more people living longer after a cancer diagnosis, improving psychological health among cancer survivors is of the utmost importance since it is linked to adherence to treatment [4] and mortality [5]. Understanding how best to improve psychological health among cancer survivors could indeed improve the quality of life among the growing number of cancer survivors as poor psychological health is common among cancer survivors [1]. Engagement in health behaviors is one way to improve the psychological health of cancer survivors [6]. However, additional research is needed to determine which health behaviors are most beneficial for psychological health among cancer survivors. The goal of the current study is to examine the association between different health behaviors and psychological health among cancer survivors.

In general, many studies and reviews have observed positive associations between various healthy lifestyle behaviors (e.g., physical activity, healthy eating, strength training) and psychological health (e.g., depression, general well-being, distress), in both clinical and nonclinical populations [7–10]. For instance, Lee and Howard [8] found increased physical activity was associated with positive emotions among older adults. Additionally, Parletta and colleagues [10] measured the effects of a Mediterranean diet intervention (e.g., high consumption of fruits, vegetables, cereals, legumes, and to a lesser extent, fish and dairy products) and found improvements in depressive symptoms over a 6-month period.

Among cancer survivors, healthy lifestyle behaviors have also been shown to be associated with psychological health. In a study of breast cancer survivors, those who engaged in more physical activity reported better health-related quality of life than survivors who were less active [11]. Hawkes and colleagues [12] conducted an intervention targeting multiple health behaviors in colorectal cancer survivors. They found distress was lower in the intervention group compared to a control group. Together, these studies demonstrate an association between healthy lifestyle behaviors and improved psychological health.

Healthy lifestyle behaviors can also be associated with another measure of psychological health: fatalistic beliefs about cancer (i.e., cancer fatalism). Cancer fatalism is the belief that there is little a person can do to prevent cancer or mitigate the health outcomes of cancer [13] and can be considered a specific type of distress. Cross-sectional studies have found that greater cancer fatalism is associated with less engagement with preventive health behaviors (e.g., lower fruit and vegetable intake) [14, 15]. Although no causal assessments have been made, it is possible that the association between cancer fatalism and healthy lifestyle behaviors is bidirectional. Cancer survivors are at risk of cancer recurrence or secondary cancer, so fears related to cancer are still relevant in the cancer survivor population. Being healthy by engaging in physical activity, having a balanced diet, and engaging in other health behaviors could reduce the belief that cancer is a potential “death sentence”, engendering a more optimistic outlook and self-efficacy in coping with the disease. Thus, in addition to examining the association between health behaviors and psychological health, it would be valuable to also better understand the association between health behavior engagement and cancer fatalism.

The purpose of this study is to examine the association between different health behaviors (i.e., fruit and vegetable consumption, sleep quality and duration, moderate physical activity [MPA], strength training, and low sedentary behavior) and psychological health (i.e., psychological distress, general health, and cancer fatalism) among cancer survivors. It is not clear if it is important to target specific health behaviors over others as a means of improving psychological health or if multiple behaviors should be targeted. In a non-cancer survivor cohort, Duncan and colleagues [16] developed an index score consisting of various unhealthy behaviors, such as smoking and physical inactivity and found individuals with higher index scores (i.e., engagement in more unhealthy behaviors) reported poorer health. A similar index could be created for healthy behaviors, such as physical activity and sleep. We hypothesized that meeting health behavior guidelines would be associated with favorable psychological health. The psychological health indicators include psychological distress, general health and cancer fatalism.

## Methods

### Participants

The data were obtained from the Health Information National Trends Survey (HINTS), a nationally representative sample of American adults that is publicly available for analysis. Data are collected annually, with questions focusing on perceptions of cancer (e.g., It seems like everything causes cancer), health behavior engagement (e.g., About how many cups of vegetables do you eat or drink each day?), health communication (e.g., Used e-mail or the Internet to communicate with a doctor or a doctor’s office), and health information technology (e.g., In the last 12 months, have you used an electronic medical device to monitor or track your health? For example a glucometer or digital blood pressure device). Participants who completed the HINTS 5 Cycle III survey were included in the study, with responses from 5438 participants. Since the focus of the investigation was on cancer survivors, only adults who answered “Yes” (*N* = 856) to the following question were actually included in the analyses: “Have you ever been diagnosed with having cancer?” See Nelson et al. [17] or the HINTS website (https://hints.cancer.gov/) for additional information on the survey instruments used and the overall methodology. Since the data are publicly available, approval from the Institutional Review Board was not required.

### Variables

The outcome variables were psychological distress, general health and cancer fatalism. The Patient Health Questionnaire-4 (PHQ-4) [18], is a 4 item self-report assessment of psychological distress related to both depression and anxiety. Higher scores suggest higher levels of psychological distress, with scores ranging from 0 to 12. There are four categories of psychological distress based on PHQ-4 score: None (0-2), Mild (3-5), Moderate (6-8), and Severe (9-12). Participants were categorized into one of the four groups, with 1 corresponding with “None” and 4 corresponding to “Severe.” General health was measured by the following question: “In general, would you say your health is …” , with participants responding on a 5-point scale from “Poor” to “Excellent.” This measure of health has been used in many previous studies, with evidence of its validity [19, 20].

Cancer fatalism was measured with three items: 1) “It seems like everything causes cancer” 2) “There’s not much you can do to lower your chances of getting cancer” and 3) “There are so many different recommendations about preventing cancer, it’s hard to know which ones to follow.” Participants responded on a 4-point scale from “Strongly agree” to “Strongly disagree” for each item. The cancer fatalism items were reverse coded so that “Strongly disagree” was 1 and “Strongly agree” was 4. Thus, higher scores on the cancer fatalism items suggested more fatalistic views about cancer. Given a relatively low Cronbach’s α of .63 and moderate inter-correlations (i.e., *r*s ranging from .31 to .42), each item was examined as a separate outcome.

The health behaviors included amount of at least moderate physical activity (MPA), strength training, fruit/vegetable consumption, amount of nightly sleep (henceforth sleep duration), sleep quality, and low sedentary behavior. Participants provided the number of days they engaged in MPA, along with the number of minutes spent engaging in MPA [21]. These two numbers were multiplied together to obtain weekly minutes engaged in MPA. Cancer survivors who exercised for 150 minutes or more a week were categorized as meeting the MPA guideline [22]. Strength training was obtained by asking participants the following: “In a typical week, outside of your job or work around the house, how many days do you do leisure-time physical activities specifically designed to strengthen your muscles?” Participants could provide “None” to “7 days.” Participants who did strength training two or more days a week were categorized as meeting the guideline [22].

For fruit and vegetable consumption, participants answered the following questions: “About how many cups of fruit (including 100% pure fruit juice) do you eat or drink each day?” and “About how many cups of vegetables (including 100% pure vegetable juice) do you eat or drink each day?” Respondents selected from the following options for both questions: “None”, “1/2 cup or less”, “1/2 cup to 1 cup”, “1 to 2 cups”, “2 to 3 cups”, “3 to 4 cups”, or “4 or more cups”. Respondents who reported eating at least “2 to 3 cups” of fruit were categorized as eating sufficient fruit. The same threshold was used for respondents who reported eating at least “2 to 3 cups” of vegetables. These thresholds were chosen according to the United States Department of Agriculture (USDA) as 1½ to 2 cups of fruit and 2 to 3 cups of vegetables are generally recommended [23, 24]. Since those who consume only 1 cup of fruit do not meet the USDA guidelines and half servings were not asked in the survey, it was decided to be more conservative and select 2 to 3 cups of fruit as the cut-off rather than 1 to 2 cups.

Participants reported their sleep quality over the past 7 days using a 4-point scale composed of “Very bad”, “Fairly bad”, “Fairly good”, and “Very good.” There are no specific guidelines for sleep quality, but for consistency with other behaviors we coded “Fairly good” or “Very good” as “good sleep quality”. For sleep duration, participants reported the average hours they slept per night over the past seven days. Participants who reported 7 to 9 hours of sleep were categorized as meeting the guideline for sleep duration [25].

Lastly, participants provided the number of hours they spent sitting. There is no established standard of what constitutes too much sedentary behavior; however, given evidence that many Americans spend half of their day or more sitting [26, 27], 8 hours was selected as the cut-off. Thus, cancer survivors who reported 8 hours or less of time spent sitting were categorized as having low sedentary levels and meeting the low sedentary “recommendation guideline”. Although physical activity and sedentary behavior are correlated, individuals who are physically active can still be too sedentary (“active couch potato”) and experience the negative effects of high levels of sedentary behavior [28]. Since sedentary behavior can be an independent predictor of health even when factoring in physical activity levels, we included it as an independent health behavior.

Basic sociodemographic/control variables were also included: age, gender, income, education, race/ethnicity, urban/rural environment, and time since cancer diagnosis. Given type of cancer can influence the psychological health of cancer survivors, we coded for cancer type: skin (non-melanoma), gynecologic, genitourinary, gastrointestinal, breast, and other. Those who had more than one cancer diagnosis were placed in a “more than one cancer diagnosis” group. Additionally, at-risk health behaviors and physical health problems are likely to be associated with both the predictors and outcomes. We included the number of comorbidities the cancer survivors had by including the following health issues: diabetes, hypertension, lung disease, obesity, and heart condition. A total score was created by summing up the number of comorbidities the cancer survivor had (i.e., scores could range from 0 to 5). We also included smoking status and alcohol consumption. Participants who answered “Every day” and “Some days” to the question “How often do you now smoke cigarettes” were considered current smokers. Participants who consumed alcohol at least once per week over the past 30 days were considered consumers of alcohol.

### Data Analysis

Sample characteristics were calculated for the demographic variables, using survey weights to estimate the national percentage. Means and standard deviations were calculated for all of the variables included in the analyses. For all seven of the health behaviors, meeting a recommended guideline was coded as 1 and not meeting the guideline was coded as 0. The health factor score was computed by adding up each of the guideline behaviors to obtain an overall health factor score with higher scores suggesting engagement in more health behaviors. Scores ranged from 0 to 7. Based on unweighted percentages, 3.1, 10.9, 20.3, 26.3, 18.7, 14.2, 4.5, and 2.0% of participants met the guideline for zero, one, two, three, four, five, six, and seven health behaviors, respectively. The weighted mean of number of guidelines met was 3.17 (SD = 1.51).

Ordinal regression was used to assess the associations between the outcome variables (psychological distress, general health, and three cancer fatalism variables) and the predictor variables (7 lifestyle behaviors as independent variables or as an index) controlling for covariates (age, gender, income, education, race/ethnicity, urban/rural environment, time since cancer diagnosis, overweight/obese, cancer type, comorbidities, smoking status, and alcohol consumption). Given our interest in assessing the associations between the individual health behaviors and the outcome variables and the composite health factor score, ten ordinal regression models were examined. In the first set of five models, each outcome variable (psychological distress, general health, and three cancer fatalism variables) was regressed on the seven health behaviors controlling for the covariates. In the second set of five models, each outcome variable was regressed on the health factor score controlling for the covariates.

It is possible that there is a point of diminishing returns with respect to the number of health behaviors in which people report engaging. In order to test for potential curvilinear relationships, the health factor score was squared and included as an additional predictor. Since the curvilinear term was added, both the health factor and health factor squared terms were mean centered when included in the analyses.

Given HINTS data are collected using a complex sampling method, analyses were conducted using weights provided by the HINTS study. The jackknife replication method was used for the weighted analyses. All descriptive statistics were conducted in R and all regression analyses were conducted in Mplus Version 8.3.

## Results

### Descriptive Statistics

Table 1 includes sample characteristics of the cancer survivors, while Table 2 includes the mean of the psychological health variables. The average age of the cancer survivors was 63.31 (SD = 16.00). The majority of cancer survivors were female (56.25%). Most cancer survivors were either overweight (38.68%) or obese (30.98%). The average time from cancer diagnosis was 12.29 (*SD* = 12.09). When examining the health behaviors guidelines, the majority of cancer survivors achieved guidelines for sleep quality, sleep duration, and low sedentary behavior, but not for the other four health behaviors. When considering the individual guidelines, 17.14% met the fruit guideline, 23.22% met the vegetable guideline, 76.60% met the sleep quality guideline, 59.32% met the sleep duration guideline, 31.85% met the MPA guideline, 31.04% met the strength training guideline, and 71.43% met the low sedentary behavior guideline.

**Table 1:** Sample characteristics of the cancer survivors

Variable	N (Unweighted)	Weighted Mean (SD) or %
**Age**		63.31 (16.00)
**Time Since Cancer Diagnosis**		12.29(12.09)
**Comorbidities**		1.49 (1.25)
**Gender**		
^a^Male	376	43.75%
Female	473	56.25%
**Race**		
^a^White cancer survivors	592	76.27%
Non-white cancer survivors	168	23.73%
**Education**		
^a^Some college or less	480	73.57%
At least college degree	371	26.43%
**Income**		
^a^$0 - $19,999	161	17.04%
$20,000 - $49,999	265	38.32%
$50,000 - $74,999	148	13.33%
$75,000 - $99,999	87	9.32%
> $100,000	184	21.99%
**Geography**		
^a^Urban	749	85.46%
Rural	107	14.54%
**BMI**		
^a^Underweight/Normal	267	30.35%
Overweight	294	38.68%
Obese	272	30.98%
**Smoking Status**		
^a^Non-Current Smoker	755	87.21%
Current Smoker	95	12.79%
**Alcohol Consumption**		
^a^Does not Consume Alcohol	408	52.68%
Consumes Alcohol	355	47.32%
**Cancer type**		
^a^Skin cancer (non-melanoma)	200	24.43%
Breast cancers	116	12.53%
Gynecologic cancers	68	8.06%
Genitourinary cancers	101	8.78%
Gastrointestinal cancers	40	4.69%
Other cancers	160	22.99%
More than one cancer	161	18.52%

^a^The reference variable in the ordinal regression models

**Table 2:** Weighted means of the Psychological Health Variables

Variable	Mean (SD)
Psychological Distress	1.50 (0.84)
General Health	3.21 (0.99)
^a^Too Many Recommendations	2.91 (0.88)
^b^Prevent Not Possible	2.07 (0.93)
^c^Everything Cause cancer	2.75 (0.95)

^a^There are so many different recommendations about preventing cancer, it’s hard to know which ones to follow

^b^There’s not much you can do to lower your chances of getting cancer

^c^It seems like everything causes cancer

### Ordinal Regression Results

Tables 3 and 4 includes the ordinal regression results (i.e., odds ratios, 95% confidence intervals, and *p*-values) of the five models that include the seven health behaviors, controlling for the covariates. As can be seen in Tables 3 and 4, individuals who met the recommended guidelines for vegetable consumption (OR = 0.43, *p* < 0.001) and sleep quality (OR = 0.54, *p* = 0.003) reported lower psychological distress scores. Individuals who met the recommended guidelines for sleep quality (OR = 1.73, *p* = 0.002) and MPA (OR = 1.88, *p* = < 0.001) reported better general health. Fruit consumption, sleep duration, strength training, and sedentary behavior were not associated with any of the psychological health measures. No significant associations were found between the health behavior guidelines and cancer fatalism, except individuals who met the recommended guidelines for MPA were more likely to report there was not much that could be done to lower one’s chances of getting cancer.

**Table 3:** Multiple ordinal regression results of each outcome variable with the health behavior guidelines

	Psychological Distress				General health				^a^Too Many Recommendations			
	OR	Low CI	High CI	*p*	OR	Low CI	High CI	*p*	OR	Low CI	High CI	*p*
^d^Female	0.77	0.49	1.20	0.246	1.31	0.89	1.91	0.167	1.01	0.66	1.54	0.956
^e^Nonwhite cancer survivor	1.09	0.72	1.66	0.688	0.70	0.49	1.00	0.051	1.06	0.57	1.96	0.852
^f^College educated	0.77	0.52	1.14	0.191	1.07	0.78	1.47	0.670	**0.63**	**0.47**	**0.84**	**0.002**
^g^$20,000 - $49,999	2.37	0.99	5.69	0.053	0.60	0.21	1.69	0.331	1.28	0.41	3.98	0.668
^g^$50,000 - $74,999	1.35	0.37	4.92	0.649	0.43	0.17	1.08	0.073	0.69	0.13	3.56	0.656
^g^$75,000 - $99,999	2.00	0.84	4.77	0.117	0.68	0.39	1.20	0.181	0.63	0.32	1.26	0.190
^g^ > $100,000	1.24	0.70	2.21	0.462	0.85	0.60	1.20	0.362	**0.70**	**0.49**	**1.00**	**0.047**
^h^Rural	1.18	0.76	1.85	0.464	0.85	0.58	1.26	0.426	1.37	0.80	2.37	0.251
^i^Overweight	0.99	0.66	1.48	0.955	1.06	0.72	1.58	0.759	1.05	0.67	1.64	0.825
^i^Obese	0.99	0.60	1.63	0.958	1.36	0.81	2.28	0.239	0.98	0.67	1.43	0.905
^j^Current smoker	**2.02**	**1.14**	**3.58**	**0.016**	0.75	0.42	1.34	0.333	0.80	0.38	1.68	0.548
^k^Consume alcohol	1.42	0.92	2.18	0.114	1.17	0.86	1.61	0.318	0.88	0.60	1.28	0.511
^l^Breast cancer	1.18	0.64	2.18	0.600	0.68	0.42	1.09	0.110	1.34	0.79	2.28	0.279
^l^Gynecologic cancers	1.14	0.56	2.33	0.718	0.90	0.55	1.47	0.667	0.84	0.41	1.75	0.649
^l^Genitourinary cancers	1.12	0.56	2.24	0.740	1.03	0.48	2.20	0.936	1.54	0.98	2.41	0.061
^l^Gastrointestinal cancers	0.84	0.33	2.15	0.714	0.82	0.34	1.98	0.659	1.25	0.57	2.73	0.575
^l^Other cancers	0.86	0.52	1.41	0.544	0.79	0.52	1.21	0.279	1.38	0.71	2.68	0.341
^l^More than one cancer diagnosis	0.84	0.49	1.46	0.542	0.79	0.49	1.27	0.331	1.36	0.76	2.45	0.298
Age	**0.98**	**0.96**	**0.99**	**0.009**	1.00	0.98	1.02	0.920	0.99	0.98	1.01	0.425
Time since cancer diagnosis	1.01	1.00	1.03	0.130	1.00	0.99	1.02	0.773	1.00	0.99	1.01	0.788
Number of comorbidities	1.12	0.92	1.37	0.253	**0.72**	**0.61**	**0.85**	**< 0.001**	1.13	0.94	1.34	0.189
Fruit	0.83	0.45	1.54	0.560	0.66	0.40	1.07	0.091	0.98	0.56	1.72	0.945
Vegetables	**0.43**	**0.26**	**0.69**	**0.000**	1.19	0.84	1.69	0.333	0.89	0.66	1.19	0.423
Sleep Quality	**0.54**	**0.35**	**0.81**	**0.003**	**1.73**	**1.21**	**2.46**	**0.003**	1.00	0.52	1.93	0.995
Sleep Duration	0.88	0.58	1.33	0.542	0.98	0.69	1.40	0.911	0.94	0.53	1.66	0.837
MPA	1.00	0.66	1.52	0.989	**1.88**	**1.35**	**2.61**	**< 0.001**	1.25	0.76	2.04	0.380
Strength training	1.21	0.82	1.80	0.336	1.39	0.94	2.06	0.104	1.00	0.68	1.49	0.984
Sedentary behavior	0.90	0.58	1.40	0.654	0.94	0.68	1.28	0.674	0.97	0.73	1.29	0.817

Note: Bolded values are significant at *p* < 0.05

^a^There are so many different recommendations about preventing cancer, it’s hard to know which ones to follow

^b^There’s not much you can do to lower your chances of getting cancer

^c^It seems like everything causes cancer

^d^reference is Male

^e^reference is White cancer survivors

^f^reference is Some college or less

^g^reference is $0 - $19,999

^h^reference is Urban

^i^reference is Underweight/Normal

^j^reference is Non-Current Smoker

^k^reference is Does not Consume Alcohol

^l^reference is Skin cancer (non-melanoma)

**Table 4:** Multiple ordinal regression results of each outcome variable with the health behavior guidelines

	^b^Prevent Not Possible				^c^Everything Cause Cancer			
	OR	Low CI	High CI	*p*	OR	Low CI	High CI	*p*
^d^Female	1.12	0.77	1.63	0.550	1.19	0.73	1.94	0.477
^e^Nonwhite cancer survivor	1.02	0.71	1.47	0.920	0.83	0.53	1.29	0.406
^f^College educated	**0.62**	**0.46**	**0.82**	**0.001**	**0.66**	**0.48**	**0.90**	**0.010**
^g^$20,000 - $49,999	2.78	0.89	8.65	0.078	1.29	0.45	3.73	0.634
^g^$50,000 - $74,999	0.97	0.32	2.93	0.964	0.68	0.32	1.47	0.330
^g^$75,000 - $99,999	1.59	0.82	3.10	0.173	1.39	0.66	2.91	0.384
^g^ > $100,000	1.06	0.68	1.64	0.808	0.72	0.47	1.09	0.116
^h^Rural	1.36	0.84	2.20	0.211	1.29	0.86	1.94	0.214
^i^Overweight	1.11	0.78	1.59	0.554	1.33	0.90	1.95	0.153
^i^Obese	1.48	0.94	2.30	0.087	1.40	0.89	2.21	0.140
^j^Current smoker	0.83	0.47	1.46	0.520	1.00	0.53	1.89	0.988
^k^Consume alcohol	0.92	0.71	1.20	0.542	1.06	0.80	1.40	0.693
^l^Breast cancer	1.09	0.61	1.94	0.781	1.26	0.70	2.24	0.440
^l^Gynecologic cancers	0.93	0.45	1.92	0.835	1.20	0.57	2.52	0.624
^l^Genitourinary cancers	**1.73**	**1.02**	**2.94**	**0.041**	1.05	0.62	1.77	0.868
^l^Gastrointestinal cancers	**2.24**	**1.18**	**4.25**	**0.013**	1.54	0.78	3.03	0.209
^l^Other cancers	0.94	0.59	1.50	0.805	0.98	0.62	1.57	0.937
^l^More than one cancer diagnosis	1.32	0.81	2.16	0.267	1.27	0.89	1.82	0.192
Age	0.99	0.98	1.01	0.345	**0.98**	**0.96**	**1.00**	**0.034**
Time since cancer diagnosis	0.99	0.98	1.01	0.309	0.99	0.98	1.01	0.206
Number of comorbidities	1.03	0.90	1.19	0.654	1.12	0.84	1.50	0.443
Fruit	0.84	0.51	1.38	0.494	0.82	0.55	1.22	0.318
Vegetables	0.93	0.65	1.35	0.715	1.45	0.96	2.19	0.081
Sleep Quality	0.91	0.61	1.35	0.631	0.78	0.52	1.17	0.228
Sleep Duration	1.06	0.79	1.41	0.706	1.02	0.77	1.35	0.894
MPA	**1.49**	**1.07**	**2.07**	**0.017**	1.07	0.74	1.55	0.710
Strength training	0.77	0.54	1.08	0.131	1.17	0.80	1.69	0.418
Sedentary behavior	1.01	0.74	1.37	0.965	1.02	0.73	1.42	0.917

Note: Bolded values are significant at *p* < 0.05

^a^There are so many different recommendations about preventing cancer, it’s hard to know which ones to follow

^b^There’s not much you can do to lower your chances of getting cancer

^c^It seems like everything causes cancer

^d^reference is Male

^e^reference is White cancer survivors

^f^reference is Some college or less

^g^reference is $0 - $19,999

^h^reference is Urban

^i^reference is Underweight/Normal

^j^reference is Non-Current Smoker

^k^reference is Does not Consume Alcohol

^l^reference is Skin cancer (non-melanoma)

Tables 5 and 6 includes the results of the five models with the overall health factor score, controlling for the covariates. As can be seen, although a higher health factor score was associated with a lower psychological distress score (OR = 0.68, *p* = 0.084), this association was not significant. The health factor score was also associated with a higher general health score (OR = 1.39, *p* = 0.055), although this association was also not significant. None of the cancer fatalism variables were significantly associated with the health factor score. However, cancer survivors with a higher health factor score were less likely to agree that there was not much that could be done to lower one’s chances of getting cancer (OR = 0.75, *p* = 0.082). None of the curvilinear associations tested were significant, which indicated there were linear rather than nonlinear associations.

**Table 5:** Multiple ordinal regression results of each outcome variable with health behavior factor score

	Psychological Distress				General health				^a^Too Many Recommendations			
	OR	Low CI	High CI	*p*	OR	Low CI	High CI	*p*	OR	Low CI	High CI	*p*
^d^Female	0.69	0.46	1.04	0.074	1.24	0.87	1.75	0.231	0.93	0.65	1.32	0.689
^e^Nonwhite cancer survivor	1.03	0.69	1.54	0.868	**0.65**	**0.45**	**0.92**	**0.016**	1.04	0.59	1.83	0.884
^f^College educated	0.75	0.52	1.07	0.111	1.08	0.79	1.46	0.633	**0.63**	**0.47**	**0.85**	**0.002**
^g^$20,000 - $49,999	2.03	0.83	4.95	0.121	0.59	0.18	1.87	0.366	1.26	0.42	3.82	0.677
^g^$50,000 - $74,999	1.30	0.34	4.96	0.702	0.45	0.18	1.13	0.088	0.67	0.13	3.39	0.626
^g^$75,000 - $99,999	1.83	0.72	4.66	0.202	0.67	0.38	1.17	0.159	0.63	0.31	1.25	0.185
^g^ > $100,000	1.30	0.74	2.31	0.364	0.85	0.58	1.25	0.419	0.73	0.51	1.06	0.095
^h^Rural	1.31	0.83	2.07	0.247	0.81	0.54	1.22	0.313	1.40	0.83	2.36	0.210
^i^Overweight	0.94	0.66	1.33	0.712	0.96	0.63	1.44	0.832	1.02	0.69	1.51	0.930
^i^Obese	0.81	0.51	1.29	0.367	1.44	0.89	2.32	0.133	0.94	0.63	1.42	0.782
^j^Current smoker	**1.80**	**1.01**	**3.22**	**0.046**	0.79	0.47	1.32	0.367	0.77	0.37	1.60	0.488
^k^Consume alcohol	1.43	0.96	2.15	0.078	1.18	0.89	1.58	0.251	0.89	0.62	1.28	0.532
^l^Breast cancer	1.23	0.68	2.21	0.488	0.70	0.46	1.07	0.096	1.36	0.82	2.26	0.236
^l^Gynecologic cancers	1.26	0.66	2.40	0.492	0.90	0.58	1.41	0.645	0.84	0.42	1.65	0.605
^l^Genitourinary cancers	1.20	0.56	2.58	0.645	1.01	0.47	2.20	0.975	1.45	0.94	2.26	0.097
^l^Gastrointestinal cancers	0.94	0.40	2.21	0.878	0.76	0.31	1.87	0.549	1.22	0.56	2.68	0.619
^l^Other cancers	0.86	0.57	1.30	0.485	0.77	0.49	1.20	0.246	1.36	0.75	2.47	0.317
^l^More than one cancer diagnosis	0.85	0.47	1.52	0.574	0.75	0.46	1.20	0.231	1.34	0.76	2.33	0.310
Age	**0.97**	**0.96**	**0.99**	**0.001**	1.00	0.99	1.02	0.645	0.99	0.98	1.01	0.493
Time since cancer diagnosis	1.01	1.00	1.03	0.161	1.00	0.99	1.01	0.858	1.00	0.99	1.01	0.720
Number of comorbidities	1.15	0.94	1.40	0.186	**0.69**	**0.60**	**0.79**	**< 0.001**	1.12	0.91	1.37	0.298
Health factor score	0.68	0.45	1.05	0.084	1.39	0.99	1.95	0.055	0.71	0.43	1.16	0.172
Health factor score squared	1.03	0.96	1.10	0.480	0.98	0.93	1.03	0.367	1.05	0.97	1.15	0.232

Note: Bolded values are significant at *p* < 0.05

^a^There are so many different recommendations about preventing cancer, it’s hard to know which ones to follow

^b^There’s not much you can do to lower your chances of getting cancer

^c^It seems like everything causes cancer

^d^reference is Male

^e^reference is White cancer survivors

^f^reference is Some college or less

^g^reference is $0 - $19,999

^h^reference is Urban

^i^reference is Underweight/Normal

^j^reference is Non-Current Smoker

^k^reference is Does not Consume Alcohol

^l^reference is Skin cancer (non-melanoma)

**Table 6:** Multiple ordinal regression results of each outcome variable with health behavior factor score

	^b^Prevent Not Possible				^c^Everything Cause Cancer			
	OR	Low CI	High CI	*p*	OR	Low CI	High CI	*p*
^d^Female	1.05	0.74	1.49	0.775	1.27	0.76	2.15	0.364
^e^Nonwhite cancer survivor	1.01	0.72	1.42	0.964	0.84	0.53	1.32	0.453
^f^College educated	**0.63**	**0.47**	**0.83**	**0.001**	**0.67**	**0.49**	**0.90**	**0.008**
^g^$20,000 - $49,999	2.51	0.96	6.55	0.059	1.28	0.45	3.62	0.642
^g^$50,000 - $74,999	0.87	0.30	2.54	0.799	0.69	0.33	1.45	0.329
^g^$75,000 - $99,999	1.49	0.80	2.80	0.209	1.40	0.69	2.83	0.355
^g^ > $100,000	1.04	0.68	1.59	0.853	0.70	0.46	1.06	0.091
^h^Rural	1.35	0.87	2.11	0.183	1.33	0.90	1.95	0.150
^i^Overweight	1.03	0.72	1.48	0.867	1.35	0.93	1.97	0.118
^i^Obese	1.40	0.90	2.19	0.135	1.39	0.87	2.23	0.171
^j^Current smoker	0.83	0.49	1.41	0.492	0.95	0.52	1.73	0.874
^k^Consume alcohol	0.97	0.75	1.25	0.800	1.09	0.82	1.44	0.570
^l^Breast cancer	1.11	0.63	1.95	0.712	1.17	0.64	2.16	0.615
^l^Gynecologic cancers	0.88	0.44	1.76	0.720	1.15	0.54	2.46	0.712
^l^Genitourinary cancers	1.62	0.92	2.87	0.096	1.05	0.61	1.79	0.860
^l^Gastrointestinal cancers	**2.16**	**1.22**	**3.84**	**0.008**	1.46	0.73	2.92	0.279
^l^Other cancers	0.91	0.59	1.41	0.671	0.92	0.60	1.40	0.692
^l^More than one cancer diagnosis	1.28	0.79	2.06	0.318	1.23	0.87	1.75	0.247
Age	1.00	0.98	1.01	0.640	**0.98**	**0.96**	**1.00**	**0.018**
Time since cancer diagnosis	0.99	0.98	1.00	0.181	0.99	0.98	1.01	0.212
Number of comorbidities	1.01	0.87	1.18	0.888	1.14	0.82	1.58	0.438
Health factor score	0.75	0.54	1.04	0.082	0.88	0.62	1.24	0.468
Health factor score squared	1.04	0.99	1.09	0.084	1.03	0.97	1.09	0.366

Note: Bolded values are significant at *p* < 0.05

^a^There are so many different recommendations about preventing cancer, it’s hard to know which ones to follow

^b^There’s not much you can do to lower your chances of getting cancer

^c^It seems like everything causes cancer

^d^reference is Male

^e^reference is White cancer survivors

^f^reference is Some college or less

^g^reference is $0 - $19,999

^h^reference is Urban

^i^reference is Underweight/Normal

^j^reference is Non-Current Smoker

^k^reference is Does not Consume Alcohol

^l^reference is Skin cancer (non-melanoma)

## Discussion

### Summary of the findings

Overall, the results show that several of the health behaviors we evaluated were associated with indicators of psychological health. Sleep quality was the only health behavior associated with multiple psychological health indicators, while vegetable consumption was only associated with psychological distress and MPA was only associated with general health. Although sleep duration was not associated with the psychological health indicators, prior research has shown sleep duration and quality are related, but distinct constructs [29]. In fact, in previous research, sleep quality has been shown to generally be a stronger predictor of psychological outcomes than sleep duration [30], which is consistent with our results.

When the health behaviors were combined to create a health factor score, the health factor score was not significantly associated with any of the indicators of psychological health included in the study. However, there were trends in the hypothesized direction for psychological distress and general health, which suggests there could be potential benefits of engaging in multiple health behaviors. However, there was no point of diminishing returns when considering a curvilinear association between the health factor score and the psychological health measures. In other words, cancer survivors can continue to reap benefits of each additional health behavior they engage in, rather than only receive marginal benefits for each health behavior. Given the cross-sectional nature of this data, however, this deserves further study within the context of a randomized controlled trial (RCT). For example, an RCT could include different conditions of varying number of health behaviors. One condition could be focused on only increasing moderate levels of physical activity among cancer survivors, while another condition focuses on increasing engagement in multiple health behaviors, such as simultaneously increasing daily vegetable intake, improving quality of sleep, and increasing moderate levels of physical activity. These conditions can be compared to a control group of cancer survivors who do not change their health behavior engagement.

When considering the health behaviors individually, health behavior engagement was not associated with cancer fatalism in these data, except for the belief that there is not much that could be done to lower one’s chances of getting cancer and MPA. Prior research has found a link between cancer fatalism and health behaviors [14, 15], however, this was observed among individuals without cancer. The null relationships between health behavior engagement and cancer fatalism here may be related to our focus on cancer survivors. Because of their experience, cancer survivors may have a different perspective of what cancer fatalism means to them. Among cancer survivors, even if they have less cancer fatalistic beliefs, they may not change their behavior. They may understand that cancer is not a death sentence or inevitable, but they may not see the point to change their behaviors if they already have cancer, even though they can get an additional diagnosis. It is unclear why those who met the MPA guideline were more likely to believe that there is not much that could be done to lower one’s chances of getting cancer. Given that it was the only health behavior to be associated with any of the cancer fatalistic variables and it was in the opposite hypothesized direction, the finding should be interpreted with caution.

### Strengths, Limitations, and Future Directions

There are several limitations that should be considered within the context of the findings. HINTS is a self-report cross-sectional study and the direction of the association (do health behaviors predict psychological health or is it vice versa) is not clear due to possible reverse causality. Self-report studies are also prone to recall bias, particularly when participants are told to recall feelings or behaviors over a period of time [31]. Of the measures included in the analyses, subjective measures of physical activity are known to have validity issues compared to objective measures, such as accelerometers [32]. Even though the dataset was nationally representative, most of the cancer survivors were 51 and older. The results may differ for a study focused on young cancer survivors. Additionally, the cancer fatalism items have been used in past research examining HINTS datasets, but there has been inconsistency in whether researchers combine these items or only use one item in their analysis [33–35].

Despite some of these limitations, there are a number of strengths. By analyzing data from a national dataset, the generalizability of the results is higher than if data were collected at only the local or regional level. We included a wide range of confounding variables (e.g., income), that otherwise could have explained the associations between the health behaviors and outcomes. Prior research has typically focused on one or two health behaviors [8–10]; we focused on seven different health behaviors in their associations with psychological health. This shows that a wide variety of health behaviors could be beneficial for health. Additionally, sleep is generally understudied compared to other health behaviors; our study included both sleep quality and duration.

While continued research is needed, there are some practical implications worth highlighting. First, there is growing interest in developing interventions to improve psychological health among cancer survivors [36]. Our findings provide evidence that health behaviors and psychological health are related, and that the number of health behaviors a cancer survivor engages in could be positively associated with psychological health. In order to lead to the best improvements in psychological health among cancer survivors, implementation scientists could develop interventions focusing on multiple health behaviors, rather than just one behavior. This also suggests that interventions should really focus on improving the sleep quality of cancer survivors. This can be done by setting up daily routines that can lead to improved sleep quality (e.g., regular sleep and wake times, reducing the use of screen time prior to bedtime).

However, care should be taken when considering the development of interventions to improve sleep quality among cancer survivors. It is possible certain cancer treatments have an influence on both psychological health and health behavior engagement [37–39], which we were not able to consider. For example, chemotherapy-induced peripheral neuropathy after treatment has been shown to be associated with less physical activity [40]. Cancer treatment has been shown to increase feelings of depression and anxiety [41, 42], which could interfere with the positive benefits of the health behaviors.

## Conclusions

Engaging in health behaviors is positively associated with better psychological health among cancer survivors. This is consistent with prior research showing the long-term benefits of health behaviors on psychological health [43, 44]. This association is not only found when considering the health behaviors individually, but also when considering the health behaviors together (although the findings are non-significant). When considering our findings at a broader level, there are implications for the development of interventions to improve psychological health among cancer survivors.

## Data Availability

The dataset analyzed during the current study is available on the NCI HINTS website, https://hints.cancer.gov/data/download-data.aspx

## Abbreviations

MPA: moderate physical activity
HINTS: Health Information National Trends Survey
PHQ-4: Patient Health Questionnaire-4
